# Nicotinic receptor activation induces NMDA receptor independent long‐term potentiation of glutamatergic signalling in hippocampal oriens interneurons

**DOI:** 10.1113/JP280397

**Published:** 2020-12-13

**Authors:** Elizabeth Nicholson, Dimitri M. Kullmann

**Affiliations:** ^1^ UCL Institute of Neurology University College London London UK

**Keywords:** interneurons, nicotinic, synaptic plasticity

## Abstract

**Key points:**

- Long‐term potentiation of glutamatergic transmission to hippocampal interneurons in stratum oriens does not require NMDA receptors and the induction mechanisms are incompletely understood.
- Extracellular stimulation, conventionally used to monitor synaptic strength and induce long‐term potentiation (LTP), does not exclusively recruit glutamatergic axons.
- We used optogenetic stimulation of either glutamatergic or cholinergic afferents to probe the relative roles of different signalling mechanisms in LTP induction.
- Selective stimulation of cholinergic axons was sufficient to induce LTP, which was prevented by chelating postsynaptic Ca^2+^ or blocking nicotinic receptors.
- The present study adds nicotinic receptors to the list of sources of Ca^2+^ that induce NMDA receptor independent LTP in hippocampal oriens interneurons.

**Abstract:**

Many interneurons located in stratum oriens of the rodent hippocampus exhibit a form of long‐term potentiation (LTP) of glutamatergic transmission that does not depend on NMDA receptors for its induction but, instead, requires Ca^2+^‐permeable AMPA receptors and group I metabotropic glutamate receptors. A role for cholinergic signalling has also been reported. However, electrical stimulation of presynaptic axons, conventionally used to evoke synaptic responses, does not allow the relative roles of glutamatergic and cholinergic synapses in the induction of LTP to be distinguished. Here, we show that repetitive optogenetic stimulation confined to cholinergic axons is sufficient to trigger a lasting potentiation of glutamatergic signalling. This phenomenon shows partial occlusion with LTP induced by electrical stimulation, and is sensitive to postsynaptic Ca^2+^ chelation and blockers of nicotinic receptors. ACh release from cholinergic axons is thus sufficient to trigger heterosynaptic potentiation of glutamatergic signalling to oriens interneurons in the hippocampus.

## Key points

- Long‐term potentiation of glutamatergic transmission to hippocampal interneurons in stratum oriens does not require NMDA receptors and the induction mechanisms are incompletely understood.
- Extracellular stimulation, conventionally used to monitor synaptic strength and induce long‐term potentiation (LTP), does not exclusively recruit glutamatergic axons.
- We used optogenetic stimulation of either glutamatergic or cholinergic afferents to probe the relative roles of different signalling mechanisms in LTP induction.
- Selective stimulation of cholinergic axons was sufficient to induce LTP, which was prevented by chelating postsynaptic Ca^2+^ or blocking nicotinic receptors.
- The present study adds nicotinic receptors to the list of sources of Ca^2+^ that induce NMDA receptor independent LTP in hippocampal oriens interneurons.

## Introduction

Many hippocampal interneurons exhibit a form of long‐term potentiation (LTP) that does not require NMDA receptors (NMDARs) for its induction (Perez *et al*. 2001; Kullmann and Lamsa, 2007; Lamsa *et al*. 2007; Galván *et al*. 2008; Pelletier and Lacaille, 2008; Oren *et al*. 2009; Sambandan *et al*. 2010; Le Duigou and Kullmann, 2011; Hainmüller *et al*. 2014). NMDAR independent LTP is especially prominent in stratum oriens interneurons, at synapses made by axon collaterals of local pyramidal neurons (Perez *et al*. 2001; Lamsa *et al*. 2007; Roux *et al*. 2013). Postsynaptic Ca^2+^ is required for LTP induction, and this can arise from several ion channels and signalling cascades, including rectifying Ca^2+^‐permeable AMPA receptors, group I metabotropic glutamate receptors (mGluRs) and voltage‐gated Ca^2+^ channels (Lamsa *et al*. 2007; Lapointe *et al*. 2004; Nicholson and Kullmann, 2014, 2017; Oren *et al*. 2009; Perez *et al*. 2001; Roux *et al*. 2013; Topolnik *et al*. 2006, 2009).

Considerable evidence also implicates acetylcholine receptors (AChRs) in NMDAR independent LTP. Both muscarinic and nicotinic receptors are expressed by stratum oriens interneurons (Lawrence *et al*. 2006; McQuiston & Madison, 1999), and at least some receive a direct cholinergic innervation from subcortical afferents (Leão *et al*. 2012). M1 muscarinic receptors share signalling mechanisms with group I mGluRs, and these receptors can partially replace one another in LTP induction (Le Duigou *et al*. 2015). Regarding nicotinic receptors, in one study, tetanic stimulation was reported only to induce LTP in oriens interneurons when nicotine was co‐applied, acting through non‐α7 nicotinic AChRs (Jia *et al*. 2010). However, another study showed that LTP induction (without agonist co‐application) was impaired in α7 knockout mice (Griguoli *et al*. 2013).

Most studies investigating LTP mechanisms in stratum oriens interneurons have used extracellular electrical stimulation of axons in the alveus or in stratum oriens; but see Alle *et al*. (2001) and Croce *et al*. (2010). Because cholinergic fibres are intermixed with glutamatergic axons, this approach does not allow the relative roles of glutamatergic and cholinergic signalling cascades to be disentangled. We therefore attempted to resolve whether nicotinic AChRs are necessary or sufficient for LTP induction by selectively expressing the optogenetic actuator channelrhodopsin‐2 (ChR2) in either glutamatergic or cholinergic axons. We developed a stimulation protocol that efficiently induces LTP when applied electrically to a mixed population of axons. The same protocol applied to the isolated glutamatergic input failed to elicit LTP, whereas, when applied to the cholinergic input, it successfully induced LTP of glutamatergic transmission. Taken together with the data obtained using pharmacological blockers of nicotinic receptors, the results suggest that Ca^2+^ influx via nicotinic receptors is sufficient for LTP induction.

## Methods

### Ethical approval

All experiments followed the Animals (Scientific Procedures) Act, 1986 and guidelines laid down by our animal welfare committee. The mice were bred in house under conditions specified in the UK Animal Welfare Act 2006 and The Welfare of Farm Animals (England) Regulations 2007, with free access to food and water.

### Surgical procedures

Male C57 mice (postnatal day 21) were anaesthetized with 5% isoflurane in a closed chamber and then transferred to a stereotaxic frame where anaesthesia was maintained with 1−2% isoflurane by mask. Breathing was continuously monitored throughout the procedure to check the depth of anaesthesia. AAV2/5‐CaMKIIa‐hChR2(H134R)‐mCherry (UNC Vector Core, Chapel Hill, NC, USA) was injected into both hemispheres of the brain. The anteroposterior injection co‐ordinate was taken as two‐thirds of the distance from bregma to lambda. The lateral co‐ordinates were 3.0 mm from the midline, and the ventral co‐ordinates were 3.5, 3.0, 2.5 and 2.0 mm from the surface of the brain, with 250 nL of adeno‐associated virus (AAV) (titre 1–8 × 10^12^ vg mL^–1^) being injected at each site. Mice were allowed to recover for at least 2 weeks prior to death.

### Brain slices

Three groups of mice were used: (i) wild type C57BL/6J mice; (ii) C57BL/6J mice that had been injected with AAV‐CaMKIIa‐hChR2(H134R)‐mCherry to express ChR2 in glutamatergic axons; and (iii) mice obtained by crossing ChAT‐IRES‐Cre and Ai32 mice (Madisen *et al*. 2012) to express ChR2 in cholinergic axons. Mice were at postnatal day (P)21–P40 at death. They were culled by cervical dislocation if aged <P30. Mice older than P30 were overdosed with i.p. pentobarbital and transcardially perfused with slicing solution. The slicing solution contained (in mm): 92 *N*‐methyl‐d‐glucamine‐Cl; 2.5 KCl; 1.25 NaH_2_PO_4_; 2 thiourea; 5 ascorbic acid; 3 Na pyruvate; 10 MgCl_2_; 25 d‐glucose; 30 NaHCO_3_; 0.5 CaCl_2_ and 1 sucrose, and was continuously gassed with 95% O_2_ and 5% CO_2_ (Ting *et al*. 2014). Horizontal 300–400 μm hippocampal slices were cut on a vibrating microtome (VT1200S, Leica Biosystems, Wetzlar, Germany; Vibroslice, Campden Instruments, Loughborough, UK) in slicing solution at 0°C. Slices were then kept at 37**°**C for 10 min and subsequently submerged at room temperature in a solution containing (in mm): 119 NaCl; 2.5. KCl; 0.5 CaCl_2_; 1.3 MgSO_4_; 1.25 NaH_2_PO_4_; 25 NaHCO_3_; and 10 glucose, gassed with 95% O_2_ and 5% CO_2_. Brain slices were shielded from light throughout the procedure.

### Electrophysiology

Slices were placed in a recording chamber mounted on an upright microscope (BX51W1; Olympus, Tokyo, Japan) and perfused with solution containing (in mm): 119 NaCl; 2.5 KCl; 2.5 CaCl_2_; 1.3 MgSO_4_; 1.25 NaH_2_PO_4_; 25 NaHCO_3_; and 10 glucose, continuously bubbled with 95% O_2_ and 5% CO_2_, at a rate of 3 mL min^–1^ at 32°C. NMDA, GABA_A_ and GABA_B_ receptors were routinely blocked with 50 μm d‐aminophosphonovalerate, 100 μm picrotoxin and 1 μm CGP 52432. Cells were visualized using infrared differential interference contrast via a ×20 0.5 NA water immersion objective. CA1 interneurons with horizontal dendrites in stratum oriens were patch clamped in whole cell mode with 4−6 MΩ resistance recording pipettes, filled with (in mm): 145 K‐gluconate; 8 NaCl; 20 KOH‐Hepes; 0.2 EGTA; and 0.5 biocytin. Current was injected, if necessary, to maintain the membrane potential between −70 and −75 mV. Hyperpolarizing and depolarizing current steps were injected to elicit a ‘sag’ potential and action potentials, and interneurons that displayed a regular firing pattern typical of oriens interneurons were selected for the experiments. Fast‐spiking interneurons, with a spike frequency >20 Hz, were rejected.

For extracellular electrical stimulation of afferents, concentric bipolar electrodes, connected to a constant current isolated stimulator (Digitimer, Welwyn Garden City, UK), were positioned in the alveus/stratum oriens border, 100–500 μm from the patched cell. The stimulus duration was 100 μs and the intensity set between 20 and 320 μA, eliciting excitatory postsynaptic potentials (EPSPs) that were subthreshold for action potential generation (baseline amplitude in the range 2–6 mV). The stimulation frequency was typically 0.03 Hz.

For optogenetic stimulation in slices from mice injected with AAV‐CaMKIIa‐hChR2(H134R)‐mCherry, we first verified expression of the fluorescent tag with epifluorescence. Excitation of ChR2 was achieved by wide field illumination delivered via a 455 nm LED (M455L2; Thorlabs, Newton, NJ, USA), controlled by a DC2100 driver (Thorlabs) coupled to the epifluorescence illuminator of the microscope with a mirror (LAS‐11‐504 MP; Laser 2000, Huntingdon, UK) in place of the dichroic. The light intensity was typically less than <1 mW mm^–2^, delivered as 1 ms pulses to elicit EPSPs in ChR2‐expressing slices. LTP induction protocols involved either pairing postsynaptic hyperpolarization for 2 min with 5 Hz stimulation (electric or light) or 1 s‐long 100 Hz stimulus trains delivered via the electrode, twice, separated by 20 s.

Data were acquired using a PCI‐6221 interface (National Instruments, Austin, TX, USA) and custom software (LabVIEW; National Instruments). Currents or voltages were low‐pass filtered (4–5 kHz), digitized at 10–20 kHz and analysed off‐line using LabVIEW and Pclamp, version 10 (Molecular Devices, San Jose, CA, USA). The initial slope of EPSPs was measured over a 2 ms window to avoid contamination by polysynaptic responses.

Slices were fixed in 4% paraformaldehyde for 12–15 h at 0–4**°**C, and then washed in PBS, and transferred to a phosphate‐buffered solution containing 0.3% triton and 0.1% streptavidin‐Alexa‐488 for 3 h at room temperature. After washing, slices were mounted with Vectashield mounting medium (Vector Laboratories, Burlingame, CA, USA). Cells were visualized and photographed using an AxioImager microscope (Carl Zeiss, Oberkochen, Germany).

### Statistical analysis

Electrophysiology data are shown as the mean ± SD. Paired or unpaired *t* tests were applied where data were normally distributed, and non‐parametric tests were applied if not. In experiments where a control pathway was not available, EPSP slopes were compared between a 5 min baseline before the plasticity protocol was applied and another period 25–30 min later. In experiments where a control pathway was available, the two pathways, each normalized by their baseline, were compared 25–30 min after the plasticity protocol was applied.

## Results

### Optogenetic stimulation of glutamatergic axons paired with hyperpolarization fails to evoke LTP

**Figure 1:**
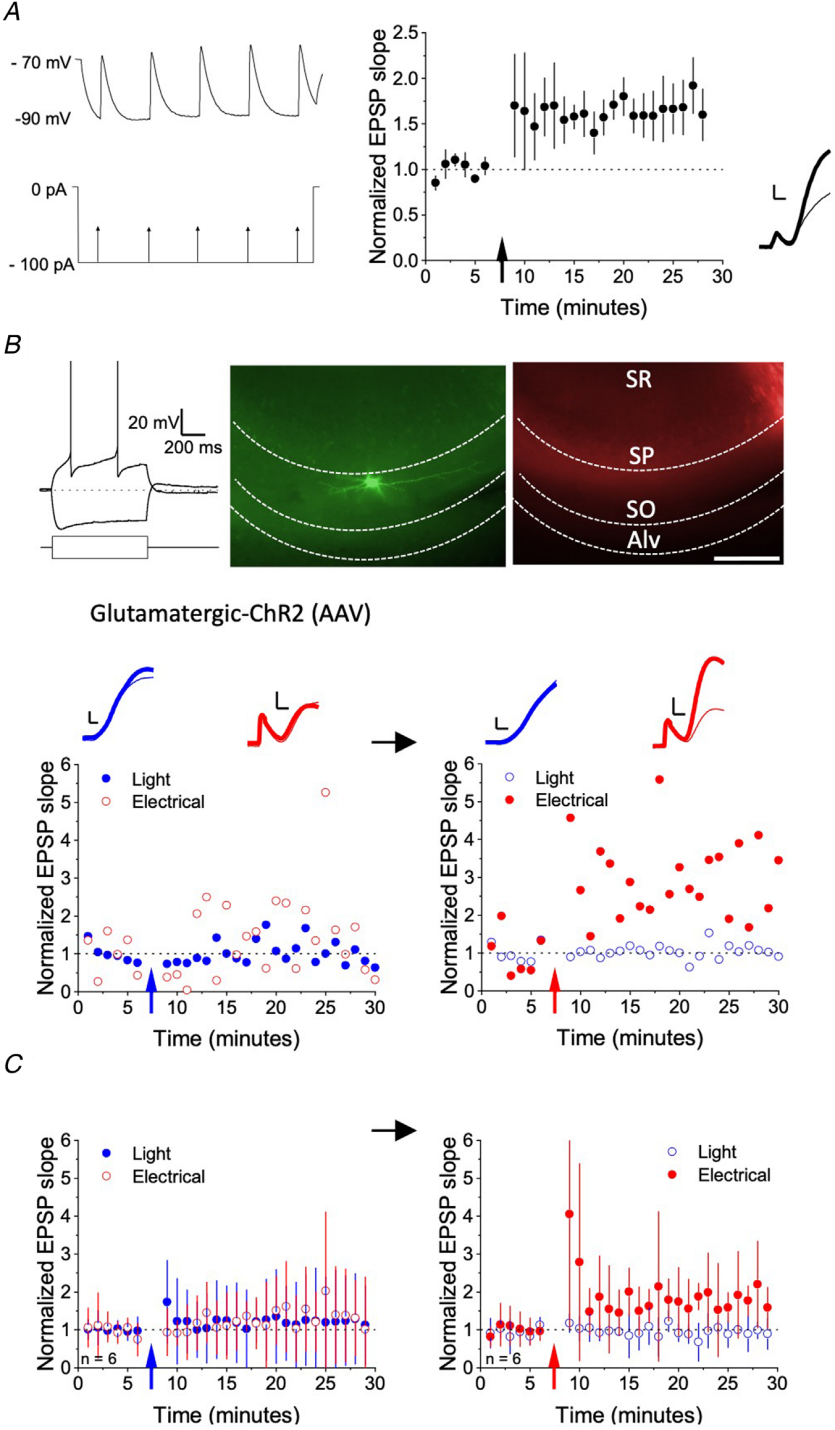
Pairing optogenetic stimulation of glutamatergic afferents with hyperpolarization fails to evoke LTP in stratum oriens interneurons *A*, left: pairing protocol. Electrical stimulation at 5 Hz (lower trace schematic) when holding the postsynaptic cell close to −90 mV (upper sample trace from one cell). Right: EPSP initial slope normalized to baseline and averaged across six cells (± SD). Inset: initial phase of EPSP in a representative example before (thin trace) and 20 min after (thick trace) pairing. Scale bars = 1 mV, 2 ms. *B*, pairing optogenetic stimulation of glutamatergic axons with hyperpolarization in an example stratum oriens interneuron. Top, left: voltage response to 100 pA positive and negative current injection. Top, middle and right: biocytin staining of the interneuron revealed with streptavidin Alexa‐488 (green), and mCherry ChR2 tag (red). The borders of the alveus (Alv), stratum oriens (SO), stratum pyramidale (SP) and stratum radiatum (SR) are indicated. Scale bar = 50 μm. Below: timecourse of EPSPs evoked by light (blue) or electrical stimulation (red). Left: the 5 Hz pairing protocol delivered to the light pathway, indicated by the blue arrow, failed to induce LTP (filled points). In the same cell, after renormalizing the baseline, the same pairing protocol delivered to the electrical pathway induced LTP (right). Traces show the averages of five trials before (thin lines) and after pairing (thick lines). Scale bars = 1 mV, 2 ms. *C*, summary plot from six cells (mean ± SD) where optogenetic (left) and electrical stimulation (right) were sequentially paired with hyperpolarization. [Color figure can be viewed at wileyonlinelibrary.com]

To isolate activity in glutamatergic axons from other afferents, we expressed mCherry‐tagged ChR2, under the CaMKIIa promoter, in pyramidal neurons of the hippocampus. ChR2 channel kinetics are not sufficiently fast to follow the 100 Hz stimulation often used to evoke LTP (Chater *et al*. 2010). We therefore initially performed a control experiment in untransduced hippocampal slices to confirm that LTP could be induced using an ‘anti‐Hebbian’ low‐frequency pairing protocol (Lamsa *et al*. 2007). NMDA, GABA_A_ and GABA_B_ receptors were routinely blocked pharmacologically. AMPA/kainate receptor‐mediated EPSPs were evoked in regular‐spiking oriens interneurons with horizontal dendrites by stimulating in the alveus via a concentric electrode at 0.03 Hz. After obtaining a baseline, alveus stimulation at 5 Hz was paired with continuous post‐synaptic hyperpolarization for 2 min, holding the cell at −90 mV by injecting negative current via the recording pipette (Lamsa *et al*. 2007; Roux *et al*. 2013). This protocol reliably induced LTP of the EPSP initial slope (178 ± 53 % of baseline, mean ± SD, *n* = 6, *P* <0.01, paired *t* test) (Fig. 1 *A*). Having confirmed that a low‐frequency pairing protocol using electrical stimulation is effective, we investigated whether LTP could be elicited when the same protocol was used with optogenetic stimulation.

EPSPs were evoked by alternating electrical and optogenetic stimuli in hippocampal slices from mice previously injected with AAV‐CaMKIIa‐hChR2(H134R)‐mCherry. We used epifluorescence to verify expression of mCherry in the alveus (Fig. 1 *B*) and focused on neurons with a sag response to hyperpolarizing current and a regular firing pattern in response to depolarizing current. Pairing 5 Hz stimulation of the optogenetic pathway with hyperpolarization to −90 mV failed to elicit LTP (Fig. 1 *B* and *C*). In the illustrated example, in the same cell, we subsequently paired electrical stimulation with the same postsynaptic hyperpolarization, using the protocol previously shown to elicit LTP in untransduced slices. Pairing electrical stimulation with postsynaptic hyperpolarization elicited a stable potentiation. When repeated in six oriens interneurons, there was no significant increase in the light‐stimulated pathway (115 ± 106%), whereas the electrically stimulated pathway increased to 172% ± 68% of baseline (*P* = 0.038, paired *t* test) (Fig. 1 *C*). The EPSP slope amplitudes were similar in both pathways (light evoked EPSP slope: 0.47 ± 0.10 mV ms^–1^, electrically evoked EPSP slope: 0.60 ± 0.36 mV ms^–1^) and the control pathways that were not stimulated during the pairing remained stable whether evoked electrically or optogenetically.

We tentatively conclude that LTP cannot reliably be elicited by glutamatergic stimulation alone, and that co‐stimulation of other axons is required to induce LTP. Cholinergic axons are a strong candidate, given the prior evidence for involvement of nicotinic receptors in LTP in oriens interneurons (Griguoli *et al*. 2013; Jia *et al*. 2010).

### Optogenetic stimulation of cholinergic fibres increases evoked EPSPs

**Figure 2:**
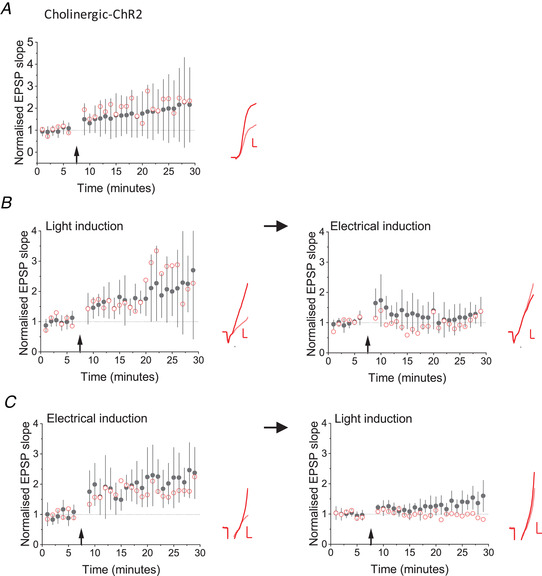
Optogenetic stimulation of cholinergic axons potentiates glutamatergic EPSPs *A*, LTP induced by pairing 5 Hz light pulses with hyperpolarization of stratum oriens interneurons in Chat‐Cre x Ai32 mice (pairing protocol indicated by arrow) in 36 cells. Black filled symbols and error bars: mean ± SD. Red open symbols: data from one cell. Inset: average EPSPs before (pink) and 25−30 min after (red) pairing. *B*, optogenetic cholinergic stimulation in Chat‐Cre x Ai32 mice (left) induced a potentiation (*n* = 9, *P* = 0.002, paired *t* test), which was followed by a smaller potentiation induced by subsequent tetanic electrical stimulation (right). Black and red symbols and traces as for (*A*). *C*, tetanic electrical stimulation in Chat‐Cre x Ai32 mice (left) led to LTP of EPSPs in oriens interneurons (*n* = 6, *P* = 0.009, paired *t* test), which was followed by a smaller effect of subsequent optogenetic cholinergic stimulation (right). Black and red symbols and traces as for (*A*). Scale bars = 2 mV, 2 ms. [Color figure can be viewed at wileyonlinelibrary.com]

To investigate the role of cholinergic axons in LTP induction more directly, we used an optogenetic strategy to stimulate them without glutamatergic axons. ChAT‐IRES‐Cre mice were crossed with Ai32 mice to allow conditional expression of ChR2‐YFP in cholinergic neurons (Hedrick *et al*. 2016). Brief pulses of blue light at an intensity and duration similar to those used to evoke glutamatergic responses in AAV‐CaMKIIa‐hChR2(H134R)‐mCherry injected animals failed to elicit a fast monosynaptic EPSP in oriens interneurons. We patched regular spiking interneurons in stratum oriens and evoked EPSPs by electrical stimulation in the alveus, and then paused stimulation for 2 min, during which 1 ms light pulses were applied at 5 Hz at resting membrane potential in current clamp. When electrical stimulation was re‐started, the EPSP initial slope showed a large increase compared to baseline (197 ± 132 %; *n* = 36), which persisted for at least 25 min (*P* < 0.001) (Fig. 2 *A*).

We looked for occlusion between LTP induced by tetanic electrical stimulation and by optogenetic activation of cholinergic afferents. When the optogenetic cholinergic protocol was delivered first, the potentiation (199 ± 101%, *n* = 9) was followed by a small LTP induced by tetanic electrical stimulation (126 ± 38%) (Fig. 2 *B*). Electrically‐induced LTP was smaller if preceded by light‐induced LTP (unpaired *t* test, *P* = 0.007). When tetanic stimulation was delivered first, LTP (229 ± 85%, *n* = 6) did not fully occlude subsequent optogenetically evoked potentiation (155 ± 52%) (Fig. 2 *C*).

### Cholinergic optogenetically‐evoked LTP requires Ca^2+^ influx and is prevented by nicotinic receptor antagonists

**Figure 3:**
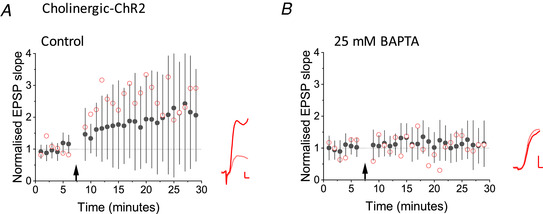
BAPTA attenuated LTP elicited by optogenetic stimulation of cholinergic axons *A*, LTP induced by optogenetic stimulation of cholinergic fibres in interleaved control experiments. Black filled symbols and error bars: mean ± SD. Red open symbols: data from one cell. Inset: average EPSPs before (pink) and 25−35 min after (red) pairing. *B*, inclusion of 25 mm BAPTA in the pipette solution profoundly attenuated LTP (*P* = 0.047, unpaired *t* test comparing potentiation elicited in both conditions). Black and red symbols and traces as for (*A*). Scale bars = 1 mV, 2 s. [Color figure can be viewed at wileyonlinelibrary.com]

The potentiation elicited by optogenetic stimulation of cholinergic fibres was abolished when 25 mm BAPTA was included in the pipette solution (114 ± 46%, *n* = 14) compared to the interleaved control experiment (222 ± 176%, *n* = 16, *P* = 0.039, Mann–Whitney) (Fig. 3). These results are consistent with Ca^2+^ influx via nicotinic receptors triggering the LTP induction cascade.

**Figure 4:**
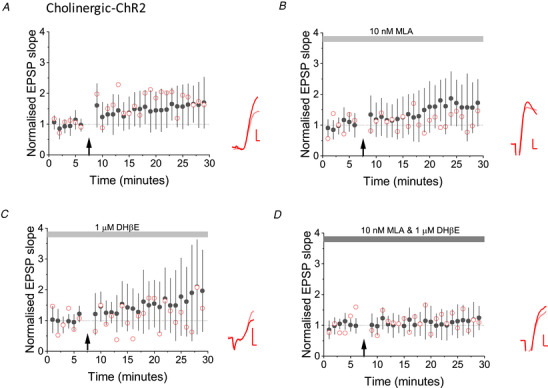
Nicotinic receptor antagonists prevented optogenetic cholinergic LTP *A*, control experiments, showing LTP induced by optogenetic stimulation of cholinergic afferents (*n* = 12, *P* = 0.01, paired *t* test). Black filled symbols and error bars: mean ± SD. Red open symbols: data from one cell. Inset: average EPSPs before (pink) and 25−30 min after (red) pairing. *B*, DHβE (1 μm) did not prevent optogenetic LTP (*n* = 8, *P* = 0.039, paired *t* test). *C*, 10 nm MLA also failed to completely block LTP (*n* = 8, *P* = 0.018, paired *t* test). *D*, LTP was blocked when both 1 mm DHβE and 10 nm MLA were bath‐applied (comparison with controls: *n* = 11, *P* = 0.009, unpaired *t* test). Scale bars = 1 mV, 2 s. Stimulation artefacts have been removed for clarity. [Color figure can be viewed at wileyonlinelibrary.com]

Finally, we investigated whether optogenetically evoked LTP in interneurons from ChAT‐Cre x Ai32 mice depends on nicotinic receptors, akin to electrically evoked LTP. In control cells LTP was again robustly induced after a period of optogenetic stimulation (160 ± 60%, *P* = 0.01) (Fig. 4 *A*). Neither 1 μm DHβE (178 ± 128%) (Fig. 4 *B*), nor 10 nm MLA (165 ± 111%) (Fig. 4 *C*) fully prevented optogenetically evoked LTP (compared to controls: DHβE *P* = 0.044, MLA *P* = 0.074). However, LTP was blocked when both 1 mm DHβE and 10 nm MLA were bath‐applied together (113 ± 47% compared to controls: *P* = 0.023) (Fig. 4 *D*).

## Discussion

The present study suggests that ACh release from cholinergic axons, leading to Ca^2+^ influx via nicotinic receptors, is sufficient to induce LTP of glutamatergic transmission to oriens interneurons. A clue with respect to the involvement of cholinergic axons came from the dissociation between the effects of optogenetic stimulation of glutamatergic axons and of extracellular electrical stimulation at 5 Hz, paired with postsynaptic hyperpolarization. Although electrical stimulation successfully evoked LTP, optogenetic glutamatergic stimulation failed. Because electrical stimulation would be expected to recruit not only glutamatergic axons, but also cholinergic axons, we repeated the experiments with ChR2 conditionally expressed in ChAT‐Cre positive neurons. Surprisingly, repetitive optogenetic stimulation alone was sufficient to induce a long‐lasting potentiation of glutamatergic transmission. This potentiation exhibited two‐way partial occlusion with electrically evoked LTP, and was prevented by postsynaptic Ca^2+^ chelation and blockade of nicotinic receptors.

The use of optogenetics as a tool to investigate LTP has previously uncovered a left‐right brain asymmetry related to differences in NMDAR composition at synapses made by CA3 afferents from either hemisphere (Kohl *et al*. 2011; Shipton *et al*. 2014) (but see also Martin *et al*. 2019). However, this asymmetry most probably does not explain the dissociation between the effects of electrical and optogenetic stimulation in the present study because AAV‐CaMKIIa‐hChR2 was injected bilaterally and NMDARs were routinely blocked.

Of note, we did not elicit robust EPSPs in oriens interneurons by optogenetic stimulation of cholinergic axons, although an increase in frequency of spontaneous glutamatergic currents was observed in some cells. This contrasts with studies that used viral expression of ChR2 to evoke monosynaptic fast optogenetic nicotinic responses in layer 1 neocortical interneurons mediated by α7 receptors (Bennett *et al*. 2012; Letzkus *et al*. 2011). Nicotinic responses mediated by either α4β2 or α7 receptors have also been reported in hippocampal interneurons using electrical or optogenetic stimulation (Alkondon *et al*. 1998; Bell *et al*. 2013; Frazier *et al*. 1998; Haam *et al*. 2018; Stone, 2007; Takács *et al*. 2018). However, we expressed ChR2 by crossing ChAT‐IRES‐Cre mice with Ai32 mice, which may have led to relatively low expression. The absence of detectable phasic nicotinic response upon stimulation is consistent with ACh acting via volume transmission, as suggested by ultrastructural evidence for non‐synaptic release sites (Descarries *et al*. 1997) (but see also Takács *et al*. 2018). Indeed, we previously reported an α7 receptor‐mediated modulation of GABA_A_ receptors in hippocampal interneurons, evoked by stimulation of cholinergic axons, despite failing to detect a clear monosynaptic nicotinic response (Wanaverbecq *et al*. 2007). The ability of cholinergic activity to trigger an intracellular cascade that modulates GABAergic transmission without a robust depolarizing response is consistent with the high Ca^2+^ permeability of α7 nicotinic AChRs and their frequent co‐location with synaptic GABA_A_ receptors (Fabian‐Fine *et al*. 2001).

Nicotinic receptors also occur presynaptically and have been implicated in the plasticity of glutamatergic transmission in many brain regions (Role & Berg, 1996; Wonnacott, 1997). However, postsynaptic Ca^2+^ chelation prevented the effect of optogenetic stimulation of cholinergic afferents on glutamatergic transmission, implying a postsynaptic induction mechanism which is consistent with previous studies of NMDAR independent LTP in hippocampal interneurons (Alle *et al*. 2001; Lamsa *et al*. 2007; Nicholson and Kullmann, 2014; Ouardouz & Lacaille, 1995). The effect of Ca^2+^ chelation does not exclude the possible involvement of astrocytic intermediaries as proposed for other actions of septal cholinergic afferents in the hippocampus (Pabst *et al*. 2016).

NMDAR independent LTP in oriens interneurons can be triggered by several sources of Ca^2+^, including Ca^2+^‐permeable AMPARs (Lamsa *et al*. 2007; Oren *et al*. 2009), group I mGluRs (Lapointe *et al*. 2004; Perez *et al*. 2001), which trigger dendritic Ca^2+^ elevation (Topolnik *et al*. 2009) and T‐type Ca^2+^ channels (Nicholson & Kullmann, 2017). Trains of postsynaptic action potentials alone, in certain circumstances, can also trigger potentiation that occludes NMDAR independent LTP (Nicholson & Kullmann, 2014). The present study adds nicotinic AChRs to the list of sources of Ca^2+^ that are able to induce synaptic potentiation. The absence of profuse dendritic spines in oriens interneurons may explain the convergence of multiple Ca^2+^ sources on the LTP induction cascade. The relative importance of each of these sources of Ca^2+^ in more physiological situations remains to be determined.

Oriens interneurons with axons that project to stratum lacunosum/moleculare (O‐LM cells) exhibit NMDAR independent LTP (Oren *et al*. 2009; Szabo *et al*. 2012), express abundant nicotinic receptors (Hagger‐Vaughan & Storm, 2019; Lawrence *et al*. 2006; Leão *et al*. 2012; McQuiston & Madison, 1999) and receive an especially large cholinergic innervation (Bell *et al*. 2013; Haam *et al*. 2018). Although we did not systematically reconstruct the axonal arborization of the oriens interneurons in the present study, their regular firing pattern and horizontally oriented dendrites suggest that at least some of them were O‐LM cells. Such interneurons have been suggested to have two developmental lineages (Chittajallu *et al*. 2013) (but see also Asgarian *et al*. 2019; Winterer *et al*. 2019) and have been shown to facilitate LTP at Schaffer collateral inputs to CA1 pyramidal cells at the same time as inhibiting LTP at the temporoammonic input, thereby affecting information flow through the hippocampal formation (Leao *et al*., 2012). The cholinergic innervation of O‐LM cells has also been implicated in modulating theta oscillations (Gu *et al*. 2020). The present study further suggests that the influence of O‐LM cells on hippocampal signalling can be persistently altered by a cholinergic afferent input. It also provides a potential mechanism by which degeneration of the cholinergic innervation of the hippocampus in Alzheimer's disease (Geula & Mesulam, 1996) interferes with information processing (Griguoli & Cherubini, 2012).

## Additional information

### Competing interests

The authors declare that they have no competing interests.

### Author contributions

All work was completed at University College London. EN and DMK conceived and designed the experiments. EN performed the experiments and analysed the data. EN and DMK interpreted the results of the experiments. EN and DMK drafted, edited and approved the final manuscript and agree to be accountable for all aspects of the work in ensuring that questions related to the accuracy or integrity of any part of the work are appropriately investigated and resolved. All persons designated as authors qualify for authorship, and all those who qualify for authorship are listed.

### Funding

This work was supported by the Wellcome Trust (095580/Z/11/Z, 212285/Z/18/Z), Medical Research Council (MR/L01095X/1) and European Research Council (232717‐INTERPLASTICITY).

## Supporting information


**Statistical Summary Document**

## Data Availability

The raw data that support the findings of this study are available from the corresponding author upon reasonable request.
